# Treatment patterns, outcomes, healthcare resource utilization, and costs associated with locally advanced squamous cell carcinoma of the head and neck in Japan

**DOI:** 10.3389/fonc.2025.1607280

**Published:** 2025-09-04

**Authors:** Kenichi Nibu, Makoto Tahara, Noriko Yoshimi, Ramzi Argoubi, Vanessa Rascon-Velasco, Makan Rahshenas, Sarah Bobiak, Ember Lu

**Affiliations:** ^1^ Department of Otolaryngology-Head and Neck Surgery, Kobe University Hospital, Kobe, Japan; ^2^ Department of Head and Neck Medical Oncology, National Cancer Center Hospital East, Kashiwa, Japan; ^3^ Oncology Medical Affairs, Merck Biopharma Co., Ltd. an affiliate of Merck KGaA, Tokyo, Japan; ^4^ Real World Evidence, Oracle, Paris, France; ^5^ Global Value Demonstration, EMD Serono Research and Development Institute, Inc. an affiliate of Merck KGaA, Billerica, MA, United States

**Keywords:** claims data, healthcare resource utilization, head and neck cancer, squamous cell carcinoma, real-world data, treatment patterns

## Abstract

**Objective:**

Treatment patterns and healthcare resource utilization (HCRU) data in patients with locally advanced (stage III to IVB) squamous cell carcinoma of the head and neck (LA SCCHN) in Japan are limited. This study describes the patient demographics and characteristics, treatment patterns, HCRU, and costs among Japanese patients with newly diagnosed LA SCCHN.

**Methods:**

This longitudinal, observational, retrospective study was conducted using real-world medical claims data from the Medical Data Vision Co., Ltd. database in Japan (1 January 2015–31 July 2022). Patients aged ≥18 years at the index date (first date of locally advanced head and neck cancer [HNC] diagnosis) and having a confirmed diagnosis of HNC during 01 January 2016–30 June 2021 in the oral cavity, larynx, hypopharynx, or oropharynx (based on ICD-10 diagnostic codes) were included. Baseline demographic and clinical characteristics were collected during the pre-index period. Treatment patterns, HCRU, and associated costs were reported during the post-index period.

**Results:**

Of the included 6741 patients with LA SCCHN, 51.3% received definitive nonsurgical treatment, 32.4% underwent primary resection, and 16.1% did not receive any agent. The most common chemotherapy agent used for chemoradiotherapy was cisplatin (74.7%). Docetaxel, cisplatin, and 5-fluorouracil combination (TPF) was used as induction chemotherapy for 28.6% of patients who received induction treatment followed by surgery and in 55.6% of patients who received induction treatment followed by radiotherapy. Patients receiving primary resection were typically older than those receiving definitive nonsurgical treatment for each cancer site and stage. Almost all patients had ≥1 all-cause hospitalizations with substantial HCRU-associated costs.

**Conclusions:**

This real-world study demonstrates that treatment of patients with LA SCCHN in Japan often included definitive nonsurgical treatment or primary surgery. The substantial burden related to LA SCCHN-associated HCRU and considerable percentage of patients receiving no treatment highlights a need for novel and effective therapies for LA SCCHN.

## 1 Introduction

Head and neck cancers (HNCs) comprise a wide range of malignant tumors originating in the upper aerodigestive tract, including the nasal cavities, paranasal sinuses, nasopharynx, hypopharynx, oropharynx, lip or oral cavity, and salivary glands (1, 2). Approximately 90% of HNCs are of squamous cell carcinoma type (3, 4) that manifest in mucosal surfaces of the oral cavity, oropharynx, and larynx (5). HNC is the 8^th^ most common cancer in the world with an annual incidence of more than 878,348 new cases in 2020 (6). It ranks 10^th^ among all cancers in Japan (7). The estimated annual incidence of HNCs (such as oral cavity, pharynx, and larynx cancer) in Japan was 29,500 cases in 2023, with a projected mortality of 9,400 deaths (7).

Men are approximately 3 times more likely to develop HNC than women in Japan (8), which is also consistent globally (9, 10). Tobacco use, alcohol consumption, and HPV infection are the most common risk factors associated with squamous cell carcinoma of the head and neck (SCCHN) development (2, 11, 12). Globally, there has been a reduction in the incidence of the larynx and nasopharyngeal cancers and an increase in the oropharynx, hypopharynx, and lip/oral cavity (13). These shifts are likely due to changes in the major risk factors for HNCs (13–15).

The prognosis and treatment of HNCs depend on patient and disease characteristics, especially tumor location, histology, and stage at diagnosis (16). Around 60% of patients with SCCHN are initially diagnosed with locally advanced disease (stage III to stage IVB) (17), resulting in a 5-year survival rate as low as 50% (18, 19).

Many patients with locally advanced squamous cell carcinoma of the head and neck (LA SCCHN) require multimodality therapy (20). Treatment approaches for SCCHN depend on whether the tumor will be removed through surgical resection (4). Standard treatment options (according to Pan-Asian adaptation of the EHNS–ESMO–ESTRO Clinical Practice Guidelines) (11) include surgery followed by adjuvant chemoradiotherapy (CRT) in approximately half of LA SCCHN patients and definitive treatment with CRT in the other half who will not undergo surgery (11). Despite the curative intent of the existing standard-of-care treatment, most patients with LA SCCHN develop local recurrence and/or distant metastasis after treatment completion (21).

The standard-of-care chemotherapy regimen for LA SCCHN is cisplatin with concurrent radiotherapy; for patients who cannot tolerate cisplatin, either a combination of carboplatin and 5-fluorouracil or cetuximab with concurrent radiotherapy are recommended as alternatives (11). Induction chemotherapy with a combination of docetaxel, cisplatin, and 5-fluorouracil (TPF) followed by radiotherapy is a standard treatment used for larynx preservation (11). However, patients of old age and those with cardiac, renal, or neurogenic dysfunction are often ineligible to receive induction with TPF combination (22). LA SCCHN disease management in Japan is unique, with induction chemotherapy being incorporated more frequently in the early stages (23).

Treatment strategies for recurrent/metastatic SCCHN have rapidly evolved, with particular emphasis on immune checkpoint inhibitors (ICIs), pembrolizumab and nivolumab (24, 25). After much initial disappointment in the locally advanced (LA) setting, ICIs have recently shown signs of efficacy in operable LA SCCHN patients, renewing interest in their potential earlier in the treatment paradigm (26, 27).

In a 2020 registry report on HNC patients in Japan, across all stages of SCCHN, the most common cancer sites were oral cavity (27.2%), hypopharynx (22.8%), oropharynx (17.5%), and larynx (16.3%) (28). It was reported that among all SCCHN patients, 55.6% of patients received primary surgery, 50.1% received radiotherapy, and 37.8% received chemotherapy; however, no description was provided by stage of disease or the specific chemotherapy agent used (28). There are no published data on treatment patterns or healthcare resource utilization (HCRU) of patients with LA SCCHN in Japan. Therefore, this real-world study aims to address this need by describing the patient profiles, treatment patterns, HCRU, and costs among newly diagnosed patients with LA SCCHN in Japan.

## 2 Materials and methods

### 2.1 Study design and data source

This longitudinal, observational, retrospective study was conducted using real-world medical claims data from the Medical Data Vision Co., Ltd. (MDV) database in Japan (1 January 2015–31 July 2022).

MDV covers nearly 45 million patients from >480 hospitals in Japan and contains anonymized administrative claims and Diagnosis Procedure Combination data of inpatients and outpatients from participating hospitals, including information on patient demographics (e.g., age, sex, height, and weight), 10^th^ edition of the International Classification of Diseases (ICD-10) diagnosis and procedure codes, laboratory tests, examinations, surgeries, treatments, and prescribed drugs (29). MDV has been commonly used for studying treatment patterns and HCRU of various cancers (30–32) including HNCs (33).

**Figure 1:**
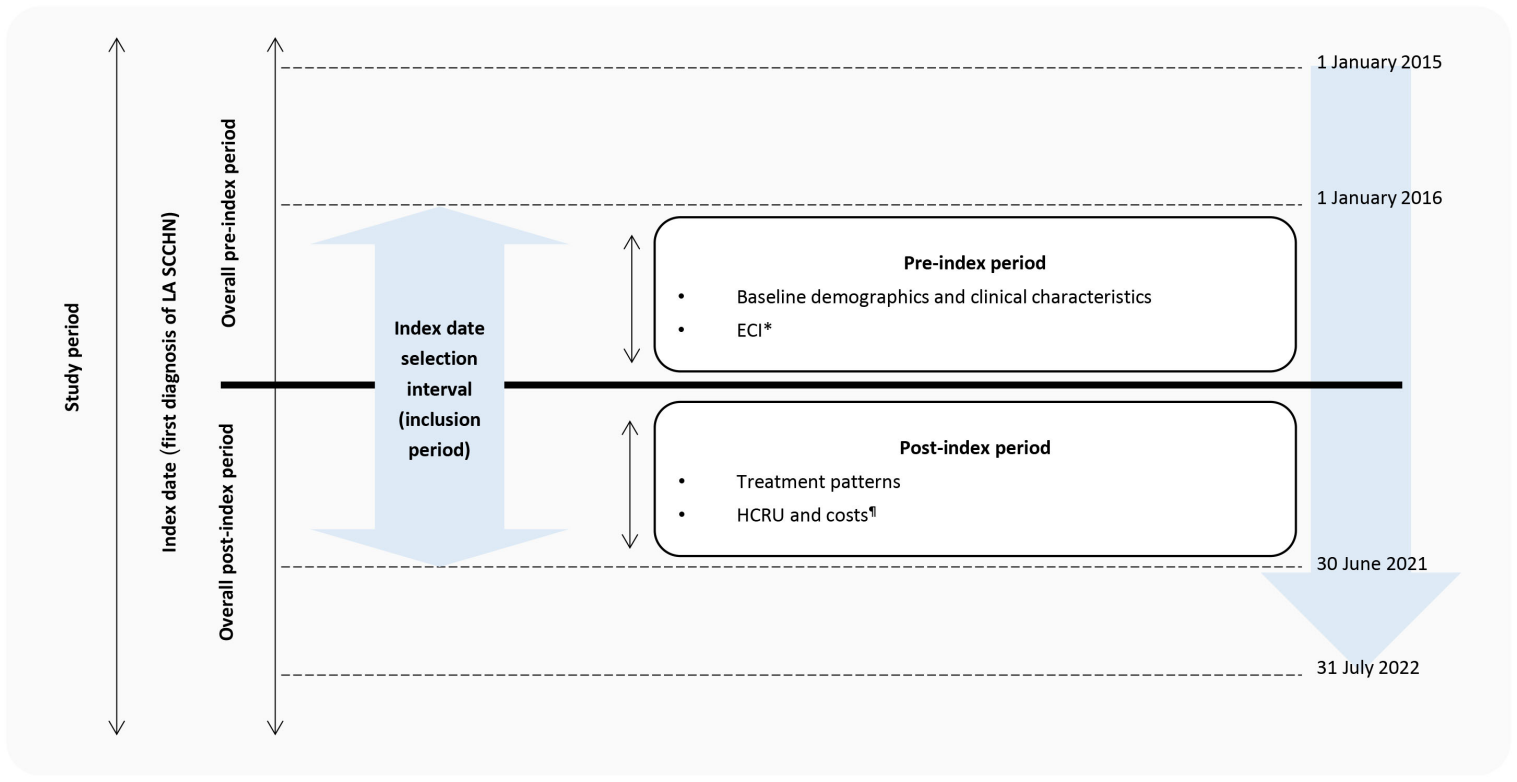
Study design. ECI, Elixhauser Comorbidity Index; HCRU, healthcare resource utilization; LA SCCHN, locally advanced squamous cell carcinoma of head and neck. *Those with at least 12 months of pre-index period. ^¶^Those with at least 12 months of post-index period.

The index date was defined as the first date of LA HNC diagnosis (based on ICD-10 codes) during the period 1 January 2016 to 30 June 2021 (**Figure 1**). The pre-index period was defined as the period of 12 months before the index date and captured patient demographics, clinical characteristics, and comorbidities. The post-index period was the follow-up period from the index date to either the end of data collection (31^st^ July 2022), loss of insurance coverage, or a record of in-hospital death, whichever came first.

This study did not require approval by an Independent Ethics Committee (IEC)/Institutional Review Board (IRB) since MDV is a fully anonymized database with no interaction/interview with any subjects. Informed consent was not required since the study utilized fully anonymized secondary data.

### 2.2 Study population

Patients were included if they: were aged ≥18 years at the index date; had a confirmed diagnosis of HNC during the period 1 January 2016 to 30 June 2021 in the oral cavity, larynx, hypopharynx, or oropharynx (defined as ≥1 ICD-10 diagnostic code of HNC in the inpatient setting or ≥2 outpatient claims with a primary ICD-10 diagnostic code of HNC [**Supplementary Table 1**]); and had valid tumor, node, and metastasis staging system (TNM) records and ≥1 claim with TNM stage III, IVA, or IVB within 60 days of HNC diagnosis.

Patients were excluded if they: were aged <18 years at the index date; had any ICD-10 code for metastatic disease prior to LA HNC diagnosis (ICD C78.x and C79.x); had TNM stage I/II/IVC prior to LA HNC diagnosis; had any other primary cancer during the pre-index period; had received chemotherapy as their only treatment for LA SCCHN (to exclude non-squamous patients [except those who also received a neck dissection]); and were participating in a clinical trial at any time during the study period.

Patients were considered treated if the first agent was initiated within 6 months following the diagnosis of LA SCCHN. The patient’s locally advanced treatment was defined by the treatment modality (surgery, radiotherapy, or systemic therapy) administered within 90 days of the start of the first treatment. In patients receiving systemic therapy as part of their treatment for LA SCCHN, any additional agents initiated within 8 days of the first administered systemic therapy were considered as part of their systemic therapy. Treatment was considered as concurrent CRT if the two treatment modalities (systemic therapy or radiotherapy) were given within 14 days of each other. A switch from one monotherapy to another monotherapy was defined by a stop of the current treatment and start of a new one after 8 days of the current treatment initiation and within 60 days of the current treatment end. A switch from one combination to another combination was defined as the removal of one component of that combination and an addition of a new one within a maximum period of 60 days, with the duration of the new combination treatment being at least 8 days. The end of LA SCCHN treatment was defined by either a switch or a discontinuation (>60-day gap between prescriptions). Adding a new treatment to the current one (add-on) was not considered as termination/advancement of a line.

### 2.3 Study measures and outcomes

The baseline demographics and clinical characteristics of patients during the pre-index period were collected, including age at index date, gender, year of the index date (2016–2021), tumor site (oral cavity, oropharynx, hypopharynx, and larynx), lymph node involvement (N0 = no lymph node involvement, N1 to N3 = lymph node involvement), tumor stage (III, IVA, IVB), body mass index (BMI), and comorbidities as Elixhauser Comorbidity Index (ECI). ECI is a point system that summarizes the overall disease burden and includes a set of 30 comorbidity measures associated with in-hospital mortality. The ECI is derived through the summation of points from each disease. The scores range from -19 (lesser disease burden) to +89 (greater disease burden) (34). This study used a coding algorithm based on ICD-10 codes (**Supplementary Table 2**) (35).

Data on treatment patterns were reported as the percentage of patients who received certain treatment sequences for LA SCCHN. Treatments for LA SCCHN were analyzed based on sub classifications as primary resection (surgery alone, surgery followed by CRT, surgery followed by radiotherapy, and chemotherapy followed by surgery), definitive nonsurgical treatment (CRT alone, radiotherapy alone, chemotherapy followed by radiotherapy, and radiotherapy followed by chemotherapy), and not treated (defined as no treatment received within 6 months of diagnosis). The treatment patterns were reported for the overall population and categorized by primary LA SCCHN treatment, primary tumor location, and the tumor stage and age of patients.

HCRU was assessed during the 12-month post-index period as the number and proportions of patients using healthcare services and represented as mean and median physician visits, all-cause hospital admissions, all-cause prescriptions (based on WHO ATC codes), laboratory tests, diagnostic imaging, surgeries (related to LA SCCHN), radiotherapy, and rehabilitation and homecare. The HCRU was reported for the overall population and categorized by LA SCCHN treatment sequences and primary tumor location.

The mean costs associated with all HCRU variables were reported in Japanese Yen during the 12-month post-index period. This estimation was based on reimbursement from the payer’s perspective. Costs per patient-year were assessed as the total sum of expenditures for patients divided by the duration (in years). The costs were reported for the overall population and categorized by LA SCCHN treatment sequences and primary tumor location.

### 2.4 Statistical analysis

The study outcomes were evaluated using descriptive analysis. Continuous variables were summarized as mean, median, standard deviation (SD), minimum, and maximum. Categorical variables were summarized as frequencies and percentages (%). Data management and analyses were performed using SAS^®^ software version 9.4.

## 3 Results

**Figure 2:**
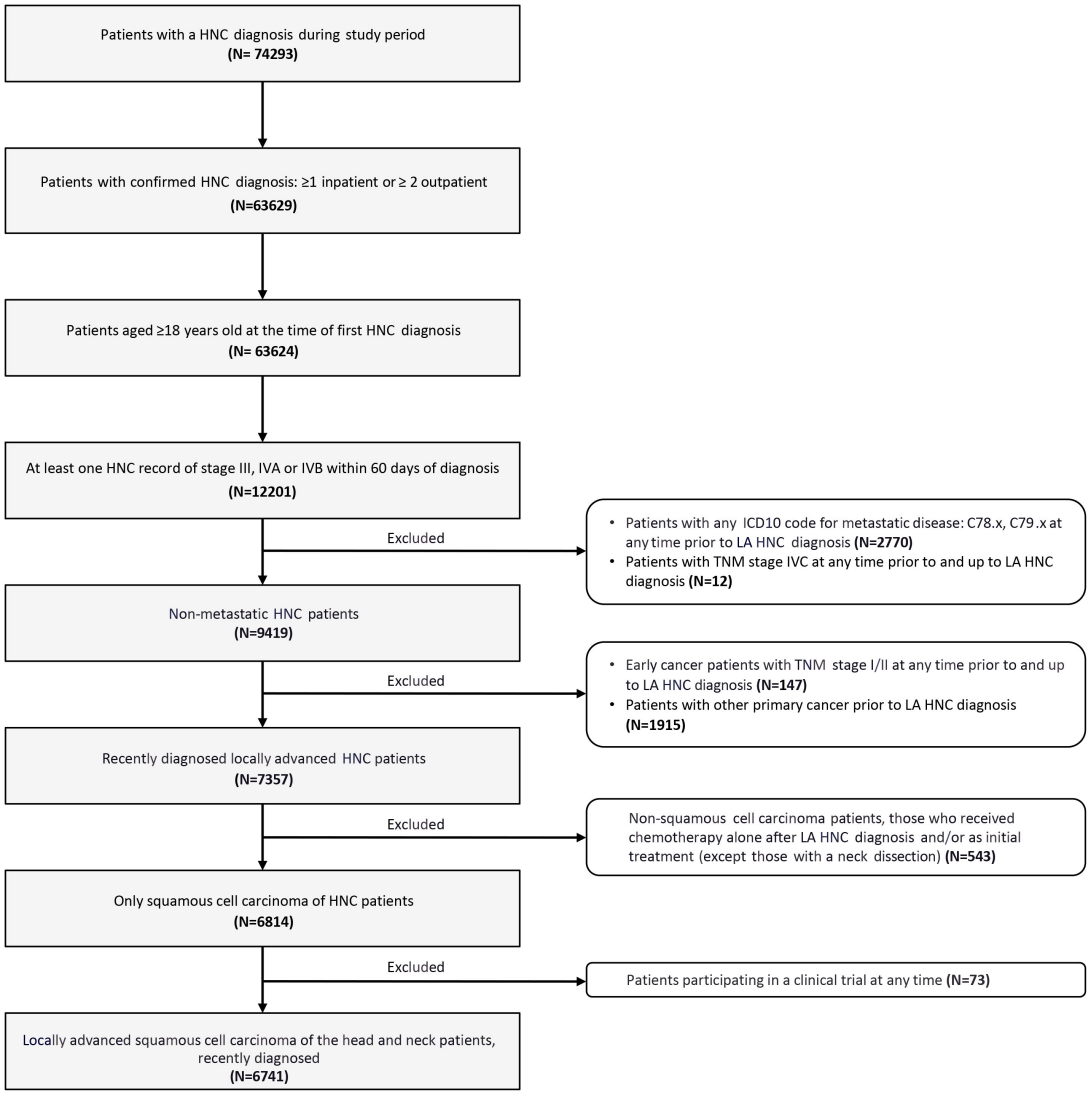
Flow diagram of patient selection. HNC, head and neck cancer; ICD, International Classification of Diseases; LA SCCHN, locally advanced squamous cell carcinoma of the head and neck; TNM, tumor, node, and metastasis staging system.

Overall, 6741 patients with newly diagnosed LA SCCHN were included in the study (**Figure 2**).

### 3.1 Demographics and clinical characteristics

Overall, the mean (SD) age of the patients was 67.7 (11.8) years at the index-date and most subjects were male (80.9%). The median follow-up duration (from index-date) was 652 days (**Table 1** **).** The distribution of primary tumor location was generally even across the oropharynx (28.6%), oral cavity (26.0%), hypopharynx (25.5%), and larynx (20.0%). At diagnosis, a higher percentage of patients were at stage IVA (53.1%) than stage III (33.6%) and IVB (13.3%). The mean (SD) ECI at baseline was 2.25 (1.5).

**Table 1:** Baseline demographic and clinical characteristics: Overall population.

Demographic characteristics	N = 6741
Age (years, at index-date)	
Mean (SD)	67.7 (11.8)
Median (Min, Max)	69 (20.0, 99.0)
Age categories (at index-date), n (%)	
≤55 years	998 (14.8)
55–65 years	1530 (22.7)
65–75 years	2501 (37.1)
>75 years	1712 (25.4)
Gender, n (%)	
Male	5451 (80.9)
Female	1290 (19.1)
Follow-up duration (days, from index-date)	
Mean (SD)	787.4 (611.7)
Median (Min, Max)	652 (1.0, 2427.0)

Clinical characteristics, n (%)	
Index year	
2016	949 (14.1)
2017	986 (14.6)
2018	1207 (17.9)
2019	1298 (19.3)
2020	1494 (22.2)
2021	807 (12.0)
Tumor site, n (%)	
Oral cavity	1749 (26.0)
Oropharynx	1925 (28.6)
Hypopharynx	1721 (25.5)
Larynx	1346 (20.0)
Lymph node involvement, n (%)	
N0	1654 (24.5)
N1	1555 (23.1)
N2	2893 (42.9)
N3	638 (9.5)
Stage at diagnosis, n (%)	
III	2266 (33.6)
IVA	3577 (53.1)
IVB	898 (13.3)
BMI categories at index date, n (%)	
Moderate/severe thinness	758 (11.2)
Underweight	649 (9.6)
Standard weight	4202 (62.3)
Overweight	988 (14.7)
Obesity	144 (2.1)
ECI at baseline	
Mean (SD)	2.25 (1.5)
Median (Min, Max)	2.0 (0.0, 12.0)
ECI Comorbidities, n (%)	
Solid Tumor without Metastasis	6581 (97.6)
Hypertension without complications	1718 (25.5)
Peptic Ulcer Disease excluding bleeding	807 (12.0)
Diabetes without complications	772 (11.5)
Liver Disease	678 (10.1)
Fluid and Electrolyte Disorders	626 (9.3)
Cardiac Arrhythmia	569 (8.4)
Chronic Pulmonary Disease	523 (7.8)
Hypothyroidism	343 (5.1)
Congestive Heart Failure	333 (4.9)

BMI, body mass index; ECI, Elixhauser Comorbidity Index; max, maximum; min, minimum; SD, standard deviation.

Patients receiving primary resection were of similar age to those receiving definitive nonsurgical treatment (mean [SD], 67.2 [12.8] *vs* 66.4 [10.7] years) (**Supplementary Table 3**). In patients receiving primary resection, the most frequent tumor location was oral cavity (49.7%) compared to the oropharynx (38.3%) in patients receiving definitive nonsurgical treatment. Initial cancer staging was IVA in approximately 50% of both subgroups. ECI at baseline was similar between the subgroups (2.3 [1.3] *vs* 2.2 [1.4]).

Patients with oropharynx cancer were the youngest (64.7 [11.1] years) while those with larynx cancer were the oldest (71.0 [9.6 years]) (**Supplementary Table 4**). The percentage of males was lower in those with oral cavity cancer (63.4%) than in oropharynx (80.2%), hypopharynx (91.3%), and larynx (91.3%) cancers. The most common stage at diagnosis was IVA in the oral cavity (56.8%), oropharynx (48.0%), and hypopharynx (62.3%) cancers, and stage III in larynx cancer (50.3%). ECI at baseline was similar between the subgroups.

### 3.2 Treatment patterns

#### 3.2.1 Treatment patterns for LA SCCHN: Overall population

Overall, 51.3% of the patients underwent definitive nonsurgical treatment, 32.4% underwent primary resection, and 16.1% were not treated with any agent within 6 months of diagnosis (**Table 2**).

**Table 2:** Treatment patterns for LA SCCHN: Overall population.

Treatment patterns, n (%)	LA SCCHN treatment N = 6741
Definitive nonsurgical treatment	3461 (51.3)
CRT alone	2655 (39.4)
Radiotherapy alone	437 (6.5)
Chemotherapy -> radiotherapy	331 (4.9)
Radiotherapy -> chemotherapy	38 (0.6)
Primary resection	2182 (32.4)
Surgery alone	1422 (21.1)
Surgery -> CRT	405 (6.0)
Surgery -> radiotherapy	242 (3.6)
Chemotherapy -> surgery	91 (1.3)
Other*	22 (0.3)
Not treated	1085 (16.1)

CRT, chemoradiotherapy; LA SCCHN, locally advanced squamous cell carcinoma of the head and neck.

**->** indicates “followed by”.

*includes surgery -> chemotherapy -> radiotherapy, chemotherapy -> surgery -> radiotherapy, and surgery -> radiotherapy -> chemotherapy.

^¶^This included n=13 patients who received chemotherapy and neck dissection with no other treatment.

#### 3.2.2 Treatment patterns for LA SCCHN: By primary LA SCCHN treatment

**Figure 3:**
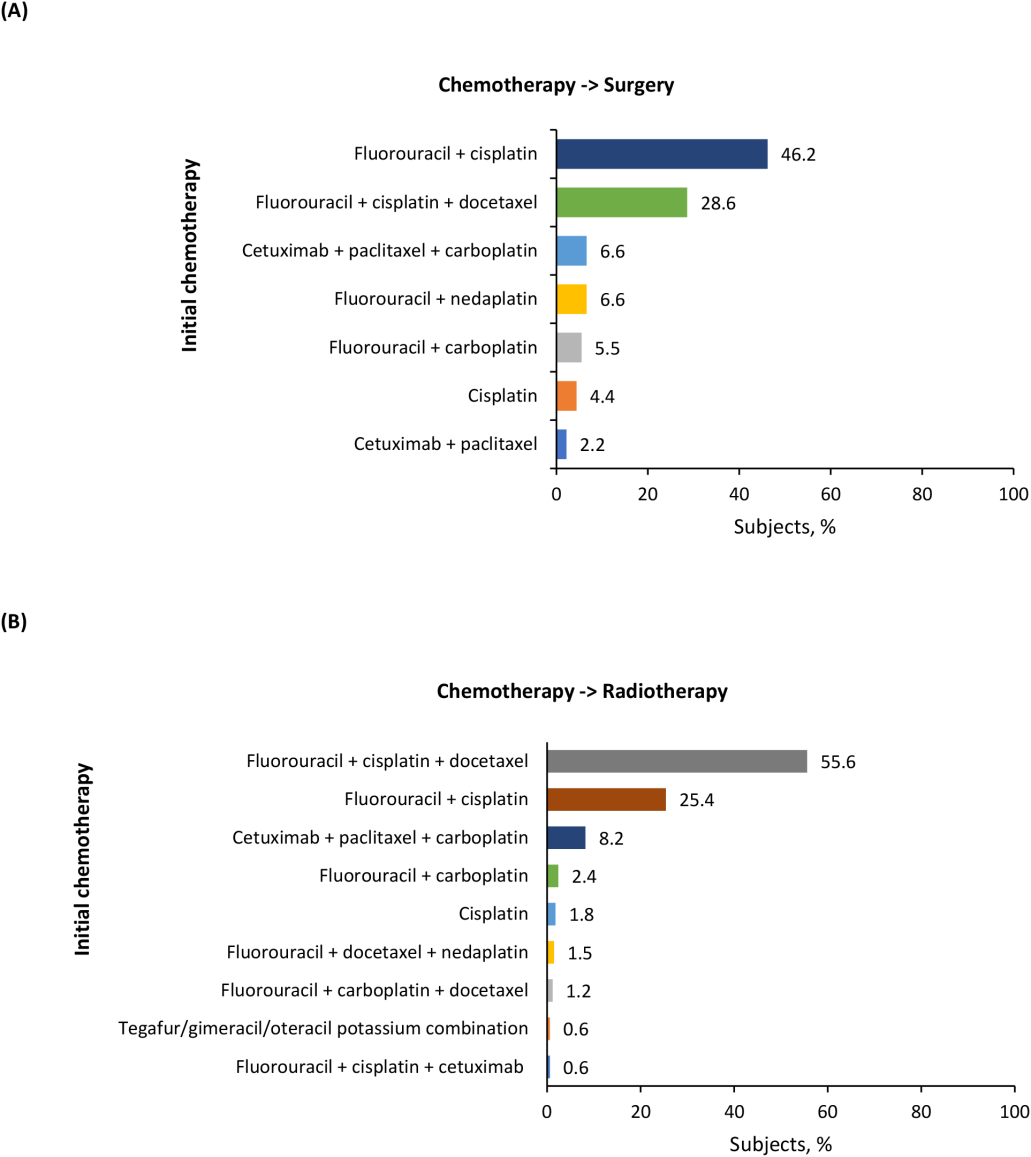
Induction chemotherapies for **(A)** chemotherapy followed by surgery and **(B)** chemotherapy followed by radiotherapy groups.

Among patients treated with primary resection, the majority underwent surgery alone (65.2%), next was surgery followed by adjuvant CRT (18.6%), surgery followed by adjuvant radiotherapy (11.1%), and induction chemotherapy followed by surgery (4.2%) (**Table 3**). In patients who received surgery followed by adjuvant CRT (n = 405), cisplatin was the most used systemic therapy component (81.7%) (**Table 3**). For patients who underwent induction chemotherapy followed by surgery (n = 91), the most frequently used chemotherapy was a combination of fluorouracil + cisplatin (46.2%), followed by TPF (28.6%), and cetuximab + paclitaxel + carboplatin (PCE) (6.6%) (**Figure 3A**). The most common chemotherapies used for induction prior to surgery based on primary tumor location are detailed in **Supplementary Table 5**.

**Table 3:** Treatment patterns for LA SCCHN: By primary treatment.

Treatment patterns		Primary resection n = 2182	Surgery -> Radiotherapy n = 242	Surgery -> CRT n = 405	Definitive nonsurgical treatment n = 3461	CRT n = 2655	Radiotherapy n = 437	Not treated n = 1085
Treatment for LA SCCHN, *n (%)*	Primary resection (n=2,182)							
	Surgery	1422 (65.2)	NA	NA	NA	NA	NA	NA
	Surgery -> CRT	405 (18.6)	NA	405 (100.0)	NA	NA	NA	NA
	Surgery -> radiotherapy	242 (11.1)	242 (100.0)	NA	NA	NA	NA	NA
	Chemotherapy -> surgery	91 (4.2)	NA	NA	NA	NA	NA	NA
	Definitive nonsurgical treatment (n=3,461)							
	CRT	NA	NA	NA	2655 (76.7)	2655 (100.0)	NA	NA
	Radiotherapy	NA	NA	NA	437 (12.6)	NA	437 (100.0)	NA
	Chemotherapy -> radiotherapy	NA	NA	NA	331 (9.6)	NA	NA	NA
	Radiotherapy -> chemotherapy	NA	NA	NA	38 (1.1)	NA	NA	NA
	Not treated	NA	NA	NA	NA	NA	NA	1085 (100.0)
Initial chemotherapy, n (%)	Cisplatin	338 (65.3)	0 (0.0)	331 (81.7)	2012 (66.5)	1983 (74.7)	0 (0.0)	12 (50.0)
	Fluorouracil + cisplatin + docetaxel	38 (7.3)	0 (0.0)	3 (0.7)	242 (8.0)	57 (2.2)	0 (0.0)	2 (8.3)
	Cetuximab (genetical recombination)	24 (4.6)	0 (0.0)	22 (5.4)	257 (8.5)	257 (9.7)	0 (0.0)	0 (0.0)
	Fluorouracil + cisplatin	58 (11.2)	0 (0.0)	12 (3.0)	215 (7.1)	128 (4.8)	0 (0.0)	1 (4.2)
	Tegafur/gimeracil/oteracil potassium combination	13 (2.5)	0 (0.0)	12 (3.0)	66 (2.2)	58 (2.2)	0 (0.0)	1 (4.2)
	Carboplatin	5 (1.0)	0 (0.0)	5 (1.2)	47 (1.6)	47 (1.8)	0 (0.0)	0 (0.0)
	Fluorouracil + carboplatin	9 (1.7)	0 (0.0)	4 (1.0)	34 (1.1)	25 (0.9)	0 (0.0)	1 (4.2)
	Cetuximab (genetical recombination) + paclitaxel + carboplatin	7 (1.4)	0 (0.0)	0 (0.0)	27 (0.9)	0 (0.0)	0 (0.0)	1 (4.2)
	Nedaplatin	8 (1.5)	0 (0.0)	8 (2.0)	28 (0.9)	28 (1.1)	0 (0.0)	0 (0.0)
	Cisplatin + docetaxel	1 (0.2)	0 (0.0)	1 (0.3)	25 (0.8)	25 (0.9)	0 (0.0)	0 (0.0)

CRT, chemoradiotherapy; LA SCCHN, locally advanced squamous cell carcinoma of the head and neck.

**->** indicates “followed by”.

In the definitive nonsurgical treatment group, the majority of patients received CRT (76.8%), next was radiotherapy alone (12.6%), and induction chemotherapy followed by radiotherapy (9.6%) (**Table 3**). In patients who received CRT (n = 2,655), cisplatin was the most commonly used systemic therapy component (74.7%) (**Table 3**). For patients who underwent induction chemotherapy followed by radiotherapy (n = 331), the most frequently used chemotherapy was TPF (55.6%), followed by fluorouracil + cisplatin (25.4%), and PCE (8.2%) (**Figure 3B**). The most common chemotherapies used for induction prior to radiotherapy based on primary tumor location are detailed in **Supplementary Table 5**.

#### 3.2.3 Treatment patterns for LA SCCHN: By primary tumor location

Primary resection was the most common in patients with oral cavity (62.1%) cancer while definitive nonsurgical treatment was the most common in patients with oropharynx (68.4%) and hypopharynx (67.2%) cancers (**Table 4**). In patients with laryngeal cancer, the percentage of those receiving definitive nonsurgical treatment (43.4%) was similar to that of primary resection (42.9%). Almost a quarter of patients with cancer of the hypopharynx (23.1%) received no treatment.

**Table 4:** Treatment patterns for LA SCCHN: By primary tumor location.

Treatment patterns, n (%)	Primary tumor location			
	Oral cavity	Oropharynx	Hypopharynx	Larynx
	n = 1749	n = 1925	n = 1721	n = 1346
Treatment sequence				
Primary resection	1079 (61.7)	363 (18.9)	150 (8.7)	568 (42.2)
Surgery	692 (39.6)	223 (11.6)	78 (4.5)	429 (31.9)
Surgery -> CRT	201 (11.5)	78 (4.1)	52 (3.0)	74 (5.5)
Surgery -> radiotherapy	137 (7.8)	36 (1.9)	16 (0.9)	53 (3.9)
Chemotherapy -> surgery	49 (2.8)	26 (1.4)	4 (0.2)	12 (0.9)
Definitive nonsurgical treatment	381 (21.8)	1324 (68.8)	1162 (67.5)	594 (44.1)
CRT	270 (15.4)	1063 (55.2)	845 (49.1)	477 (35.4)
Radiotherapy	83 (4.8)	131 (6.8)	151 (8.8)	72 (5.4)
Chemotherapy -> radiotherapy	21 (1.2)	121 (6.3)	154 (9.0)	35 (2.6)
Radiotherapy -> chemotherapy	7 (0.4)	9 (0.5)	12 (0.7)	10 (0.7)
Not treated	283 (16.2)	229 (11.9)	398 (23.1)	175 (13.0)

CRT, chemoradiotherapy.

**->** indicates “followed by”.

#### 3.2.4 Treatment patterns for LA SCCHN: By tumor stage and age of patients

Among all patients with stage III, IVA, and IVB, 39.1%, 31.3%, and 19.6%, respectively, had primary resection, while 50.5%, 50.1%, and 58.1%, respectively, underwent definitive nonsurgical treatment (**Table 5**). For each cancer site and stage, patients who had primary resection were typically older than patients who received definitive nonsurgical treatment. With increasing disease severity, definitive nonsurgical treatment became the treatment of choice for an increasing proportion of patients with cancer of the oral cavity (III to IVB, 17.3% to 39.8%) while primary resection decreased (III to IVB, 82.7% to 60.2%). Patients with cancers of the larynx and hypopharynx were generally older than patients with cancer of the oral cavity and oropharynx.

**Table 5:** Treatment patterns for LA SCCHN: By tumor stage and age.

Stage at diagnosis	III n = 2266			IVA n = 3577			IVB n = 898		
Treatment	n (%)	Age, years		n (%)	Age, years		n (%)	Age, years	
		Mean	Median		Mean	Median		Mean	Median
Definitive nonsurgical treatment	1144 (50.5)	66.0	67.0	1795 (50.2)	66.4	67.0	522 (58.1)	67.0	68.0
Radiotherapy	127 (5.6)	76.5	78.0	234 (6.5)	75.8	78.0	76 (8.5)	76.2	77.0
CRT	954 (42.1)	64.8	67.0	1326 (37.1)	65.2	67.0	375 (41.8)	65.8	67.0
Chemotherapy -> Radiotherapy	55 (2.4)	63.0	64.0	213 (5.9)	63.3	64.0	63 (7.0)	62.9	62.0
Radiotherapy -> Chemotherapy	8 (0.4)	66.3	65.5	22 (0.6)	69.2	70.0	8 (0.9)	67.1	67.5
Primary resection	885 (39.1)	67.3	70.0	1121 (31.3)	67.1	69.0	176 (19.6)	67.4	69.0
Surgery	668 (29.5)	68.0	70.0	694 (19.4)	68.8	70.0	60 (6.7)	72.0	74.0
Surgery -> CRT	112 (4.9)	63.4	66.0	223 (6.2)	61.5	64.0	70 (7.8)	61.9	64.0
Surgery -> Radiotherapy	72 (3.2)	68.0	70.0	135 (3.8)	69.1	72.0	35 (3.9)	71.1	73.0
Surgery -> Chemotherapy -> Radiotherapy	6 (0.3)	63.5	61.5	2 (0.1)	61.0	61.0	2 (0.2)	63.0	63.0
Surgery -> Radiotherapy -> Chemotherapy	0 (0.0 )	.	.	4 (0.1)	67.5	69.5	0 (0.0)	.	.
Chemotherapy -> Surgery	27 (1.2)	63.8	68.0	55 (1.5)	63.5	66.0	9 (1.0)	66.7	67.0
Chemotherapy -> Surgery -> Radiotherapy	0 (0.0)	.	.	8 (0.2)	68.8	69.5	0 (0.0)	.	.
Chemotherapy	2 (0.1)	61.0	61.0	7 (0.2)	69.7	70.0	4 (0.4)	68.5	71.5
Not treated	235 (10.4)	72.8	75.0	654 (18.3)	73.4	73.0	196 (21.8)	71.9	73.0
Primary tumor location*									
Oral cavity									
Definitive nonsurgical treatment	80 (17.3)	63.2	67.0	229 (27.9)	66.4	69.0	72 (39.8)	66.4	70.0
Primary resection	383 (82.7)	66.1	69.0	593 (72.1)	65.6	68.0	109 (60.2)	67.2	70.0
Oropharynx									
Definitive nonsurgical treatment	503 (73.0)	63.1	64.0	633 (79.6)	64.9	66.0	188 (90.4)	65.4	66.0
Primary resection	186 (27.0)	61.4	61.5	162 (20.4)	64.4	65.0	20 (9.6)	65.5	68.0
Hypopharynx									
Definitive nonsurgical treatment	217 (84.1)	68.4	69.0	717 (88.5)	67.0	67.0	228 (91.9)	68.1	69.0
Primary resection	41 (15.9)	70.9	71.0	93 (11.5)	69.0	68.0	20 (8.1)	70.9	72.0
Larynx									
Definitive nonsurgical treatment	344 (55.6)	69.4	70.0	216 (44.2)	68.7	69.5	34 (55.7)	68.7	70.0
Primary resection	275 (44.4)	72.3	73.0	273 (55.8)	71.3	71.0	27 (44.3)	67.4	68.0

CRT, chemoradiotherapy.

^*^Percentages are calculated excluding the “Not-treated” group.

-> indicates “followed by”.

### 3.3 HCRU

During the 12-month post-index period, overall, 94.1% of patients had a physician visit with a median of 15 visits per patient (**Table 6**). Almost all patients (99.8%) reported ≥1 all-cause hospitalization and all-cause prescriptions, with a median of 2 hospitalizations and 351 prescriptions, respectively. Almost all patients (99.1%) had lab testing performed with a median of 28 tests per patient, and 85.5% of patients had imaging conducted with a median of 3 images per patient. Over half of patients (55.1%) received rehabilitation and homecare with a median of 17 visits per patient.

**Table 6:** Healthcare resource utilization in the 12-month post-index period: By LA SCCHN treatments.

Characteristics		All patients n = 5284	Treatment						
			Primary resection n = 1809	Surgery -> Radiotherapy n = 198	Surgery -> CRT n = 354	Definitive nonsurgical treatment n = 2851	CRT n = 2236	Radiotherapy n = 290	Not treated n = 613
Physician visits overall	n (%)	4970 (94.1)	1781 (98.5%)	194 (98.0)	354 (100.0)	2782 (97.6)	2194 (98.1)	267 (92.1)	396 (64.6)
	Mean* (SD)	18.9 (12.5)	18.1 (12.7)	31.6 (19.6)	19.4 (12.3)	20.1 (12.4)	19.4 (11.5)	21.1 (15.2)	13.8 (10.9)
	Median* (Min, Max)	15 (1, 119)	14 (1, 88)	32 (2, 88)	16 (1, 67)	17 (1, 70)	16 (1, 70)	16 (1, 68)	12 (1, 119)
All-cause hospital admissions	n (%)	5273 (99.8)	1809 (100.0)	198 (100.0)	354 (100.0)	2851 (100.0)	2236 (100.0)	290 (100.0)	602 (98.2)
	Mean* (SD)	2.2 (1.6)	2.1 (1.5)	2.1 (1.3)	2.9 (1.9)	2.4 (1.6)	2.3 (1.6)	2 (1.4)	1.5 (1.1)
	Median* (Min, Max)	2 (1, 23)	2 (1, 23)	2 (1, 9)	2 (1, 23)	2 (1, 19)	2 (1, 19)	2 (1, 12)	1 (1, 10)
All-cause prescriptions	n (%)	5273 (99.8)	1809 (100.0)	198 (100.0)	354 (100.0)	2851 (100.0)	2236 (100.0)	290 (100.0)	602 (98.2)
	Mean* (SD)	466.4 (410.7)	422.5 (405.4)	435.3 (402.5)	546.5 (341.4)	528.2 (404.8)	521.2 (397.8)	474.2 (432.6)	300.8 (389.3)
	Median* (Min, Max)	351 (1, 5221)	300 (18, 5221)	315 (58, 3022)	464 (51, 2771)	417 (10, 3652)	409 (23, 3652)	340.5 (10, 2687)	183 (1, 3219)
Laboratory tests	n (%)	5237 (99.1)	1802 (99.6)	198 (100.0)	354 (100.0)	2850 (99.9)	2235 (99.9)	290 (100.0)	574 (93.6)
	Mean* (SD)	37 (33.1)	35.2 (33.3)	40.1 (42.5)	46.6 (33.8)	41.8 (32.6)	41.7 (31.2)	31.7 (27.7)	18.2 (26.1)
	Median* (Min, Max)	28 (1, 347)	26 (1, 264)	23 (1, 261)	35 (7, 216)	32 (1, 275)	32 (1, 238)	22 (1, 153)	10 (1, 347)
Diagnostic imaging	n (%)	4519 (85.5)	1592 (88.0)	181 (91.4)	318 (89.8)	2504 (87.8)	1975 (88.3)	244 (84.1)	414 (67.5)
	Mean* (SD)	3.5 (2.1)	3.7 (2.2)	3.8 (2.3)	4.2 (2.3)	3.6 (2.0)	3.5 (2.0)	3.5 (2.1)	2.7 (1.9)
	Median* (Min, Max)	3 (1, 18)	3 (1, 18)	3 (1, 18)	4 (1, 15)	3 (1, 13)	3 (1, 12)	3 (1, 13)	2 (1, 15)
Rehabilitation and homecare	n (%)	2912 (55.1)	1267 (70.0)	135 (68.1)	231 (65.3)	1350 (47.4)	1004 (44.9)	179 (61.7)	286 (46.7)
	Mean* (SD)	22.3 (21.8)	22.9 (21.0)	24.7 (25.1)	29.3 (22.4)	21.7 (20.9)	21.4 (20.3)	26.2 (24.4)	22.1 (28.5)
	Median* (Min, Max)	17 (1, 241)	18 (1, 219)	18 (1, 163)	24 (1, 117)	16 (1, 191)	16 (1, 173)	20 (1, 191)	14 (1, 241)

CRT, chemoradiotherapy; LA SCCHN, locally advanced squamous cell carcinoma of the head and neck; max, maximum; min, minimum; SD, standard deviation.

-**>** indicates “followed by”.

n (%) indicates the number and percentage of patients in each group.

*indicates mean/median number of overall physician visits, all-cause hospital admissions, all-cause prescriptions, laboratory tests, diagnostic imaging, and rehabilitation and homecare received in patients with LA SCCHN.

Patients who underwent primary resection had numerically lower physician visits (median of 14 *vs* 17 visits per patient, respectively) and all-cause prescriptions (median of 300 *vs* 417 prescriptions per patient, respectively) than patients who received definitive non-surgical treatment. Rehabilitation and homecare were reported by a higher percentage of patients who had primary resection (70.0%) than those receiving definitive nonsurgical treatment (47.4%), with median values of 18 and 16 visits per patient, respectively.

Across all tumor locations, almost all patients (>99.5%) reported ≥1 all-cause hospitalization and ≥1 all-cause prescriptions; the median physician visits/patient ranged between 14–16 (**Supplementary Table 6**). Patients with hypopharynx cancer had higher number of all-cause prescriptions (median, 430 prescriptions) than those with other with cancers (315–339 prescriptions). Surgeries related to LA SCCHN as well as rehabilitation and homecare were reported by a higher percentage of patients with oral cavity cancer (71.3% and 66.0%, respectively), while radiation therapy was reported by a higher percentage of patients with oropharynx (81.6%) and hypopharynx (80.1%) cancers.

### 3.4 HCRU-associated costs

Overall, the cost per patient-year of major healthcare expenditure in the 12-month post-index period were attributed to: physician visits, ¥123,200; all-cause hospitalizations, ¥1,363,327; all-cause prescriptions, ¥239,214; lab tests, ¥28,504; diagnostic imaging, ¥1,684; and rehabilitation and homecare, ¥19,321 (**Table 7**). Patients undergoing primary resection had numerically lower costs (per patient-year) than those receiving definitive nonsurgical treatment for physician visits (¥108,532 *vs* ¥138,149) and all-cause prescriptions (¥193,143 *vs* ¥283,771); but had higher all-cause hospitalization costs (¥1,413,811 *vs* ¥1,324,291).

**Table 7:** Healthcare costs (JPY, ¥) in the 12-month post-index period: By LA SCCHN treatments.

Characteristics		All patients n = 5284	Treatment						
			Primary resection n = 1809	Surgery -> Radiotherapy n = 198	Surgery -> CRT n = 354	Definitive nonsurgical treatment n = 2851	CRT n = 2236	Radiotherapy n = 290	Not treated n = 613
Physician visits overall	n (%)	4970 (94.1)	1781 (98.5)	194 (98.0)	354 (100.0)	2782 (97.6)	2194 (98.1)	267 (92.1)	396 (64.6)
	Mean (SD), ¥	346,183 (346,821.8)	306,812.3 (350,774.4)	723,650.9 (583,800.3)	369,814.3 (355,836.9)	390,490.8 (349,200.4)	373,504.9 (318,510.4)	420,844.5 (477,203.1)	211,659.5 (242,300.8)
	Total sum of expenditures, ¥	1,720,529,663.0	546,432,791.0	140,388,266.0	130,914,256.0	1,086,345,451.0	819,469,784.0	112,365,487.0	83,817,171.0
	Cost per patient-year, ¥	123,200.0	108,531.6	269,633.9	139,054.6	138,149.3	131,254.7	168,836.0	80,701.1
All-cause hospital admissions	n (%)	5273 (99.8)	1809 (100.0)	198 (100.0)	354 (100.0)	2851 (100.0)	2236 (100.0)	290 (100.0)	602 (98.2)
	Mean (SD), ¥	3,610,720.9 (2,515,486.5)	3,934,896.5 (2,402,291.1)	4,152,289 (2,440,100.6)	5,209,806.8 (2,326,529.8)	3,652,629.4 (2,499,356.2)	3,659,638.5 (2,514,139.5)	3,148,623 (2,203,572.1)	2,389,949.3 (2,543,174.3)
	Total sum of expenditures, ¥	19,039,331,347.0	7,118,227,689.0	822,153,221.0	1,844,271,596.0	10,413,646,539.0	8,182,951,744.0	913,100,662.0	1,438,749,487.0
	Cost per patient-year, ¥	1,363,327.3	1,413,810.6	1,579,051.9	1,958,949.9	1,324,291.3	1,310,665.4	1,371,989.3	1,385,261.9
All-cause prescriptions	n (%)	5273 (99.8)	1809 (100.0)	198 (100.0)	354 (100.0)	2851 (100.0)	2236 (100.0)	290 (100.0)	602 (98.2)
	Mean (SD), ¥	633,549.4 (1,465,043.7)	537,552.4 (972,837.6)	509,828.7 (850,139.7)	887,765.4 (1,274,534.2)	782,690.4 (1,808,604.4)	807,859.4 (1,930,568.2)	497,952.3 (1,022,822.0)	204,887.8 (344,429.0)
	Total sum of expenditures, ¥	3,340,705,853.0	972,432,269.0	100,946,089.0	314,268,961.0	2,231,450,396.0	1,806,373,711.0	144,406,177.0	123,342,444.0
	Cost per patient-year, ¥	239,214.0	193,142.9	193,880.1	333,810.5	283,770.9	289,327.3	216,979.1	118,757.0
Laboratory tests	n (%)	5237 (99.1)	1802 (99.6)	198 (100.0)	354 (100.0)	2850 (99.9)	2235 (99.9)	290 (100.0)	574 (93.6)
	Mean (SD), ¥	76,010.9 (59,283.6)	73,957.2 (57,276.7)	80,726.5 (62,837.5)	95,926.2 (64,566.0)	83,322.7 (60,055.5)	82,488.5 (58,601.9)	72,303.2 (60,015.8)	44,593 (48,073.0)
	Total sum of expenditures, ¥	398,068,940.0	133,270,810.0	15,983,840.0	33,957,870.0	237,469,680.0	184,361,820.0	20,967,940.0	25,596,370.0
	Cost per patient-year, ¥	28,504.1	26,470.0	30,699.0	36,069.4	30,198.7	29,529.3	31,505.6	24,644.8
Diagnostic imaging	n (%)	4519 (85.5)	1592 (88.0)	181 (91.4)	318 (89.8)	2504 (87.8)	1975 (88.3)	244 (84.1)	414 (67.5)
	Mean (SD), ¥	5,203.7 (3,686.1)	5,353.3 (3,701.0)	5,606.6 (4,038.1)	6,387.7 (3,874.5)	5,342.7 (3,730.9)	5,319.4 (3,671.1)	4,756.6 (3,475.7)	3,795.9 (2,945.9)
	Total sum of expenditures, ¥	23,515,600.0	8,522,400.0	1,014,800.0	2,031,300.0	13,378,000.0	10,505,900.0	1,160,600.0	1,571,500.0
	Cost per patient-year, ¥	1,683.9	1,692.7	1,949.1	2,157.6	1,701.3	1,682.7	1,743.9	1,513.1
Rehabilitation and homecare	n (%)	2912 (55.1)	1267 (70.0)	135 (68.1)	231 (65.3)	1350 (47.4)	1004 (44.9)	179 (61.7)	286 (46.7)
	Mean (SD), ¥	92,657.8 (127,475.4)	103,337.2 (137,215.8)	108,627.3 (124,592.6)	126,510.1 (127,129.4)	80,682.8 (95,237.0)	78,612.6 (91,727.9)	101,832.4 (112,454.6)	100,449.6 (193,896.7)
	Total sum of expenditures, ¥	269,819,648.0	130,928,210.0	14,664,690.0	29,223,830.0	108,921,748.0	78,927,050.0	18,228,000.0	28,728,590.0
	Cost per patient-year, ¥	19,320.7	26,004.7	28,165.4	31,041.0	13,851.5	12,641.8	27,388.7	27,660.6

CRT, chemoradiotherapy; JPY, ¥, Japanese Yen; LA SCCHN, locally advanced squamous cell carcinoma of the head and neck; max, maximum; min, minimum; SD, standard deviation.

**->** indicates “followed by”.

Patients with primary hypopharynx cancer had higher costs than other primary cancers (oral cavity, oropharynx, and larynx) for physician visits (¥150,785), all-cause prescriptions (¥310,874), and radiation therapy (¥246,794) (**Supplementary Table 7**). Patients with oral cavity cancer had higher all-cause hospitalization costs (¥1,634,627) and costs for surgeries related to LA SCCHN (¥174,664) than those with other cancers.

## 4 Discussion

This observational retrospective study using the MDV claims database in Japan assessed patient demographics and characteristics, treatment patterns, HCRU, and costs in Japanese patients with LA SCCHN. Among treatments for LA SCCHN, definitive nonsurgical treatment was more common than primary resection; however, this is largely dependent on the primary tumor location. Cisplatin was the most common systemic agent used in CRT. Healthcare costs were high and were mainly attributed to all-cause hospitalizations and all-cause prescriptions.

The characteristics of the MDV database makes it especially well-suited and representative of LA SCCHN patients in Japan. Our study population was predominantly male (80.9%), and more than half of the patients were ≥65 years of age, which is consistent with the latest Japanese epidemiological data (8, 28). The most common primary tumor locations for HNC were the oropharynx and oral cavity, similar to the trend observed in previous studies (36–38). More than half of this study population (~53%) had IVA stage disease at diagnosis, which is also consistent with previous research in LA SCCHN (38). This may be attributed to late diagnosis due to the lack of specificity of the symptoms and routine screening methods (39).

Patients with LA SCCHN require a multidisciplinary approach to treatment, considering patient-specific factors and complexities of the treatments (4, 40). HNC treatment is challenging since it involves several critical structures and varying radiosensitivity of nearby tissues (41). Ideally, surgery followed by adjuvant (chemo)radiotherapy or definite nonsurgical treatment with primary CRT is the treatment modality of choice for locally advanced disease (11). Surgery may not be a treatment option in certain cases, such as when the tumor is unresectable due to its location, the patient prefers not to undergo surgery, or the patient’s health conditions preclude surgery (4). In this study, the proportion of patients with LA SCCHN receiving definitive nonsurgical treatment (51.3%) was higher than primary surgical resection (32.4%), with the tumor location being a key driver for the choice of treatment. While cancers of the oral cavity were commonly treated surgically, cancers of the oropharynx and hypopharynx were mostly treated with definitive nonsurgical treatment. Our findings are in line with evidence supporting definitive nonsurgical treatment approaches (primary radiotherapy or CRT) as effective alternatives in patients ineligible for surgery. Additionally, these approaches may be appropriate for patients with resectable tumors that can be treated adequately without surgery, such as in cases of laryngeal preservation (4, 11). Overall, 16.1% of the patients in this study did not receive any treatment, which aligns with a real-world data analysis of LA SCCHN treatment patterns in the US (16% untreated) (38).

Induction chemotherapy with TPF is a standard treatment recommended for larynx preservation (11). Our study demonstrates its substantial use in the real-world in patients undergoing surgery as well. Induction chemotherapy has been shown to help in guiding the selection of subsequent therapy in LA SCCHN (i.e., chemo-selection) (42–44). A study by Lee et al. has suggested that response to induction chemotherapy can be used to select a definitive locoregional treatment; in patients responding to induction chemotherapy, either surgery or definitive concurrent CRT is considered appropriate, while in patients not responding to induction chemotherapy, a multi-disciplinary approach is needed to obtain optimal outcomes (42). Further, PCE as induction treatment is used as an alternative to TPF in daily clinical practice in Japan, as reported in several studies (22, 44, 45). Induction with PCE has been shown to have comparable efficacy and lower toxicity than induction with TPF (22, 44). In our study, TPF was the induction chemotherapy of choice in 28.6% of patients who received induction chemotherapy followed by surgery and in 55.6% of patients who received induction chemotherapy followed by radiotherapy; however, the use of induction PCE was much lower (6.6% and 8.2%, respectively). These numbers, though lower than expected, are unsurprising as data for PCE usage were only available near the end of the study period.

Cisplatin based CRT was the most common chemotherapy in the overall study population as well in the subgroups of primary resection and definitive nonsurgical treatment cohorts. This is in line with the current standard-of-care chemotherapy regimens for LA SCCHN (11). The treatment strategy for resectable LA SCCHN has not changed till very recently, with emerging data suggesting a potential role for ICIs. In contrast, there has been little to no progress in treatment options for unresectable LA SCCHN, despite substantial research efforts (46, 47). Meanwhile, the therapeutic landscape for recurrent/metastatic SCCHN has shifted over the last decade towards less toxic alternatives, such as ICIs (48, 49).

Studies reporting the HRCU burden in patients with LA SCCHN, specifically in Japan, are scarce. In this study, during the 12-month post-index period, HCRU for the overall study population was high with almost all patients having a hospital admission and several hundred all-cause prescriptions. More than half of the study population had received rehabilitation and homecare, further demonstrating the demanding resource intensity of treating LA SCCHN. The use of rehabilitation and home care services was higher in those undergoing primary resection than those receiving definitive non-surgical treatment. This trend may be attributed to the differences in post-treatment outcomes between the two treatment types in patients with LA SCCHN. Across all tumor locations, almost all patients reported at least one hospital admission and several all-cause prescriptions, thus highlighting the burden of LA SCCHN on healthcare use, irrespective of the tumor location. Radiation therapy was reported by a higher percentage of patients with oropharynx and hypopharynx cancers than those with other cancers, while surgeries related to LA SCCHN were reported by a higher percentage of patients with oral cavity cancer, further emphasizing that the tumor location is a key driver for the choice of treatment in LA SCCHN.

Globally, healthcare costs associated with the management of LA SCCHN are substantial, with most studies reporting only direct medical costs of HNC (50). Over the 12-month post-treatment period, the HCRU-associated costs were considerable and attributed mainly to all-cause hospitalizations, followed by all-cause prescriptions, and physician visits. Patients with oral cavity cancer had higher all-cause hospitalization costs and costs for surgeries related to LA SCCHN than in other cancers, as expected, considering that primary resection was the most common treatment in patients with oral cavity cancer. Our findings are similar to previous studies that have reported that healthcare costs among cancer patients are driven by hospitalization costs (50, 51). The comparison of cost estimates among different studies is challenging due to the heterogeneity in definitions of the disease, as well as the methods, and data sources used (52). Further, national insurance schemes differ across countries.

The interpretation of this study’s results should take into account the strengths and limitations of both the data source and study design. Firstly, these findings may not be representative of hospitals that have not adopted the Diagnosis Procedure Combination system. Nevertheless, based on existing epidemiological data, the demographics and characteristics of the study population appear aligned with the general population with LA SCCHN in Japan. Secondly, as MDV data is captured separately from each contracted hospital, we are unable to track patient movement between hospitals, which may lead to missing data during the follow-up period. However, patients in Japan prefer to continue receiving care at their initial hospital. Data on histology, and HPV status were not available. Though this study did not examine survival outcomes, HPV status substantially impacts prognosis, specifically in patients with oropharyngeal cancers, and thus would ultimately influence HCRU and related costs. In order to exclude patients with non-squamous cell carcinoma, we excluded patients who received only chemotherapy (unless they also received a neck dissection); however, this also excluded patients who received induction chemotherapy prior to CRT. The study duration overlapped with the COVID-19 pandemic. Lastly, there may be a slight lag between real-world practice changes and their reflection in the database.

This real-world study provided valuable insights into the LA SCCHN population in Japan using data from the MDV database. The study results demonstrated the unique nature of treatments used in patients with LA SCCHN in Japan. The primary treatment modality is largely driven by the tumor location. Cisplatin remains the standard-of-care chemotherapy agent used. Uniquely in Japan, we observed a higher utilization of induction chemotherapy prior to surgery or radiotherapy. Further, patients incurred substantial burden related to HCRU and costs, and a considerable percentage of patients did not receive any treatment for LA SCCHN. This study underscores the slow progress in treatment advancements over the past two decades and highlights the ongoing need for novel and effective therapies for patients with both operable and inoperable LA SCCHN.

## Data Availability

The data analyzed in this study was obtained from MDV Co. Ltd. Japan, and is not publicly available for patient privacy and confidentiality reasons. Requests to access these datasets should be directed to Ramzi Argoubi, ramzi.argoubi@oracle.com.
