# Supplementary material for: Treatment patterns, outcomes, healthcare resource utilization, and costs associated with locally advanced squamous cell carcinoma of the head and neck in Japan
Source: Front Oncol. 2025 Sep 4;15:1607280. doi: 10.3389/fonc.2025.1607280 (PMC12444664; doi:10.3389/fonc.2025.1607280)
Supplement: Supplementary file 1 [file DataSheet1.docx]

# **Supplementary material**

## SUPPLEMENTARY TABLE 1 ICD-10 codes for selected SCCHN cancers.

| **Site/cancer type** | **ICD-10 code** | **Diagnosis** |
| --- | --- | --- |
| **Oral cavity** | | |
| Malignant neoplasm of lip | C00.3 | Upper lip, inner aspect |
|  | C00.4 | Lower lip, inner aspect |
|  | C00.5 | Lip, unspecified, inner aspect |
| Malignant neoplasm of other and unspecified parts of tongue | C02.0 | Dorsal surface of tongue |
|  | C02.1 | Border of tongue |
|  | C02.2 | Ventral surface of tongue |
|  | C02.3 | Anterior two-thirds of tongue, part unspecified |
|  | C02.4 | Lingual tonsil |
|  | C02.8 | Overlapping lesion of tongue |
|  | C02.9 | Tongue, unspecified |
| Malignant neoplasm of gum | C03.0 | Upper gum |
|  | C03.1 | Lower gum |
|  | C03.9 | Gum, unspecified |
| Malignant neoplasm of floor of mouth | C04.0 | Anterior floor of mouth |
|  | C04.1 | Lateral floor of mouth |
|  | C04.8 | Overlapping lesion of floor of mouth |
|  | C04.9 | Floor of mouth, unspecified |
| Malignant neoplasm of palate | C05.0 | Hard palate |
|  | C05.1 | Soft palate |
|  | C05.2 | Uvula |
|  | C05.8 | Overlapping lesion of palate |
|  | C05.9 | Palate, unspecified |
| Malignant neoplasm of other and unspecified parts of mouth | C06.0 | Cheek mucosa |
|  | C06.1 | Vestibule of mouth |
|  | C06.2 | Retromolar area |
|  | C06.8 | Overlapping lesion of other and unspecified parts of mouth |
| **Oropharynx** | | |
| Malignant neoplasm of base of tongue | C01 | Malignant neoplasm of base of tongue |
| Malignant neoplasm of tonsil | C09.0 | Tonsillar fossa |
|  | C09.1 | Tonsillar pillar (anterior)(posterior) |
|  | C09.8 | Overlapping lesion of tonsil |
|  | C09.9 | Tonsil, unspecified |
| Malignant neoplasm of oropharynx | C10.0 | Vallecula |
|  | C10.1 | Anterior surface of epiglottis |
|  | C10.2 | Lateral wall of oropharynx |
|  | C10.3 | Posterior wall of oropharynx |
|  | C10.4 | Branchial cleft |
|  | C10.8 | Overlapping lesion of oropharynx |
|  | C10.9 | Oropharynx, unspecified |
| Malignant neoplasm of other and ill-defined sites in the lip, oral cavity and pharynx | C14.2 | Waldeyer ring |
| **Hypopharynx** |  |  |
| Malignant neoplasm of hypopharynx | C12 | Malignant neoplasm of piriform sinus |
|  | C13.0 | Post cricoid region |
|  | C13.1 | Aryepiglottic fold, hypopharyngeal aspect |
|  | C13.2 | Posterior wall of hypopharynx |
|  | C13.8 | Overlapping lesion of hypopharynx |
|  | C13.9 | Overlapping lesion of hypopharynx |
| **Larynx** |  |  |
| Malignant neoplasm of larynx | C32.0 | Glottis |
|  | C32.1 | Supraglottis |
|  | C32.2 | Subglottis |
|  | C32.3 | Laryngeal cartilage |
|  | C32.8 | Overlapping lesion of larynx |
|  | C32.9 | Larynx, unspecified |

ICD, International Classification of Diseases; SCCHN, squamous cell carcinoma of the head and neck.

## SUPPLEMENTARY TABLE 2 ICD-10 codes for Elixhauser Comorbidity Index.

| **Elixhauser Comorbidities** | **ICD-10 codes** | **Weights (van Walraven**  **Algorithm)** |
| --- | --- | --- |
| Congestive heart failure | I09.9, I11.0, I13.0, I13.2, I25.5, I42.0, I42.5-I42.9, I43.x, I50.x, P29.0 | 7 |
| Cardiac arrhythmias | I44.1-I44.3, I45.6, I45.9, I47.x-I49.x, R00.0, R00.1, R00.8, T82.1, Z45.0, Z95.0 | 5 |
| Valvular disease | A52.0, I05.x-I08.x, I09.1, I09.8, I34.x-I39.x, Q23.0, Q23.3, Z95.2, Z95.4 | −1 |
| Pulmonary circulation Disorders | I26.x, I27.x, I28.0, I28.8, I28.9 | 4 |
| Peripheral vascular disorders | I70.x, I71.x, I73.1, I73.8, I73.9, I77.1, I79.0, I79.2, K55.1, K55.8, K55.9, Z95.8, Z95.9 | 2 |
| Hypertension, uncomplicated | I10.x | 0 |
| Hypertension, complicated | I11.x-I13.x, I15.x | 0 |
| Paralysis | G04.1, G11.4, G80.1, G80.2, G81.x, G82.x, G83.0- G83.4, G83.9 | 7 |
| Other neurological disorders | G10.x-G13.x, G20.x-G22.x, G25.4, G25.5, G31.2,  G31.8, G31.9, G32.x, G35.x-G37.x, G40.x, G41.x, G93.1, G93.4, R47.0, R56.x | 6 |
| Chronic pulmonary | I27.8, 127.9, J40.x-J47.x, J60.x-J67.x, J68.4, J70.1, J70.3 | 3 |
| Diabetes, uncomplicated | E10.0, E10.1, E10.9, E11.0, E11.1, E11.9, E12.0,  E12.1, E12.9, E13.0, E13.1, E13.9, E14.0, E14.1, E14.9 | 0 |
| Diabetes, complicated | E10.2-E10.8, E11.2-E11.8, E12.2- E12.8, E13.2- E13.8, E14.2-E14.8 | 0 |
| Hypothyroidism | E00.x-E03.x, E89.0 | 0 |
| Renal failure | I12.0, I13.1, N18.x, NI9.x, N25.0, Z49.0-Z49.2, Z94.0, Z99.2 | 5 |
| Liver disease | B18.x, I85.x, I86.4, I98.2, K70.x, K71.1, K71.3-  K71.5, K71.7, K72.x-K74.x, K76.0, K76.2- K76.9. Z94.4 | 11 |
| Peptic ulcer disease excluding bleeding | K25.7, K25.9, K26.7, K26.9, K27.7, K27.9, K28.7, K28.9 | 0 |
| AIDS/HIV | B20.x-B22.x, B24.x | 0 |
| Lymphoma | C81.x-C85.x, C88.x, C96.x, C90.0, C90.2 | 9 |
| Metastatic cancer | C77.x-C80.x | 12 |
| Solid tumor without metastasis | C00.x-C26.x, C30.x-C34.x, C37.x-C41.x, C43.x, C45.x-C58.x, C60.x-C76.x, C97.x | 4 |
| Rheumatoid arthritis/ collagen vascular diseases | L94.0, L94.1, L94.3, M05.x, M06.x, M08.x, M12.0,  M12.3, M30.x, M31.0-M31.3, M32.x-M35.x, M45.x, M46.1, M46.8, M46.9 | 0 |
| Coagulopathy | D65-D68.x, D69.1, D69.3-D69.6 | 3 |
| Obesity | E66.x | −4 |
| Weight loss | E40.x-E46.x, R63.4, R64 | 6 |
| Fluid and electrolyte disorders | E22.2, E86.x, E87.x | 5 |
| Blood loss anemia | D50.0 | −2 |
| Deficiency anemia | D50.8, D50.9, D51.x-D53.x | −2 |
| Alcohol abuse | F10, E52, G62.1, I42.6, K29.2, K70.0, K70.3,  K70.9, T51.x, Z50.2, Z71.4, Z72.1 | 0 |
| Drug abuse | F11.x-F16.x, F18.x, F19.x, Z71.5. Z72.2 | −7 |
| Psychoses | F20.x, F22.x-F25.x, F28.x, F29.x, F30.2, F31.2, F31.5 | 0 |
| Depression | F20.4, F31.3-F31.5, F32.x, F33.x, F34.1, F41.2, F43.2 | −3 |

ICD, International Classification of Diseases.

## SUPPLEMENTARY TABLE 3 Demographic and clinical characteristics: By LA SCCHN treatment sequences.

| Characteristics | Treatment | | | | | | |
| --- | --- | --- | --- | --- | --- | --- | --- |
|  | **Primary resection** | Surgery -> Radiotherapy | Surgery -> CRT | **Definitive nonsurgical treatment** | CRT | Radiotherapy | **Not treated** |
|  | n = 2182 | n = 242 | n = 405 | n = 3461 | n = 2655 | n = 437 | n = 1085 |
| Age (years, at index-date) | | | | | | | |
| Mean (SD) | 67.2 (12.8) | 69.0 (12.3) | 62.1 (11.8) | 66.4 (10.7) | 65.1 (10.1) | 76.1 (9.8) | 73.0 (11.4) |
| Median (Min, Max) | 69 (20.0, 96.0) | 71 (21.0, 90.0) | 64 (20.0, 85.0) | 67 (21.0, 99.0) | 67 (21.0, 92.0) | 78 (42.0, 99.0) | 74 (24.0, 99.0) |
| Age categories (at index-date) | | | | | | | |
| ≤55 | 379 (17.4) | 35 (14.5) | 106 (26.2) | 534 (15.4) | 444 (16.7) | 19 (4.4) | 85 (7.8) |
| 55–65 | 449 (20.6) | 42 (17.4) | 114 (28.2) | 930 (26.9) | 760 (28.6) | 42 (9.6) | 146 (13.5) |
| 65–75 | 770 (35.3) | 82 (33.9) | 147 (36.3) | 1343 (38.8) | 1098 (41.4) | 113 (25.9) | 380 (35.0) |
| >75 | 584 (26.8) | 83 (34.3) | 38 (9.4) | 654 (18.9) | 353 (13.3) | 263 (60.2) | 474 (43.7) |
| Gender, n (%) | | | | | | | |
| Male | 1638 (75.1) | 171 (70.7) | 320 (79.0) | 2924 (84.5) | 2242 (84.4) | 356 (81.5) | 877 (80.8) |
| Female | 544 (24.9) | 71 (29.3) | 85 (21.0) | 537 (15.5) | 413 (15.6) | 81 (18.5) | 208 (19.2) |
| Follow-up duration (from index-date) |  |  |  |  |  |  |  |
| Overall, Mean (SD) | 872.5 (587.4) | 825.1 (547.7) | 874.8 (552.1) | 860.6 (598.3) | 887.8 (586.5) | 603.7 (561.4) | 381.9 (538.1) |
| Median (Min, Max) | 737.5 (4.0, 2421.0) | 714.0 (69.0, 2269.0) | 738.0 (73.0, 2385.0) | 729.0 (3.0, 2427.0) | 768.0 (5.0, 2427.0) | 441.0 (3.0, 2315.0) | 91.0 (1.0, 2416.0) |
| Clinical Characteristics | | | |  |  |  |  |
| Index Date, n (%) |  |  |  |  |  |  |  |
| 2016 | 313 (14.3) | 26 (10.7) | 51 (12.6) | 484 (14.0) | 354 (13.3) | 63 (14.4) | 151 (13.9) |
| 2017 | 292 (13.4) | 32 (13.2) | 54 (13.3) | 500 (14.5) | 371 (14.0) | 64 (14.7) | 192 (17.7) |
| 2018 | 397 (18.2) | 45 (18.6) | 66 (16.3) | 588 (17.0) | 462 (17.4) | 72 (16.5) | 218 (20.1) |
| 2019 | 415 (19.0) | 49 (20.3) | 73 (18.0) | 677 (19.6) | 522 (19.7) | 81 (18.5) | 204 (18.8) |
| 2020 | 492 (22.6) | 55 (22.7) | 107 (26.4) | 783 (22.6) | 615 (23.2) | 103 (23.6) | 216 (19.9) |
| 2021 | 273 (12.5) | 35 (14.5) | 54 (13.3) | 429 (12.4) | 331 (12.5) | 54 (12.4) | 104 (9.6) |
| Tumor site, n (%) |  |  |  |  |  |  |  |
| Oral cavity | 1085 (49.7) | 137 (56.6) | 201 (49.6) | 381 (11.0) | 270 (10.2) | 83 (19.0) | 283 (26.1) |
| Oropharynx | 368 (16.9) | 36 (14.9) | 78 (19.3) | 1324 (38.3) | 1063 (40.0) | 131 (30.0) | 229 (21.1) |
| Hypopharynx | 154 (7.1) | 16 (6.6) | 52 (12.9) | 1162 (33.6) | 845 (31.8) | 151 (34.6) | 398 (36.7) |
| Larynx | 575 (26.4) | 53 (21.9) | 74 (18.3) | 594 (17.2) | 477 (18.0) | 72 (16.5) | 175 (16.1) |
| Lymph node involvement, n (%) | |  |  |  |  |  |  |
| N0 | 793 (36.3) | 58 (24.0) | 78 (19.3) | 562 (16.2) | 429 (16.2) | 92 (21.1) | 298 (27.5) |
| N1 | 524 (24.0) | 58 (24.0) | 72 (17.8) | 843 (24.4) | 695 (26.2) | 94 (21.5) | 187 (17.3) |
| N2 | 732 (33.6) | 101 (41.7) | 194 (47.9) | 1673 (48.3) | 1266 (47.7) | 198 (45.3) | 481 (44.4) |
| N3 | 133 (6.1) | 25 (10.3) | 61 (15.1) | 383 (11.1) | 265 (10.0) | 53 (12.1) | 118 (10.9) |
| Stage at diagnosis, n (%) | |  |  |  |  |  |  |
| III | 885 (40.6) | 72 (29.8) | 112 (27.7) | 1144 (33.1) | 954 (35.9) | 127 (29.1) | 235 (21.7) |
| IVA | 1121 (51.4) | 135 (55.8) | 223 (55.1) | 1795 (51.9) | 1326 (49.9) | 234 (53.6) | 654 (60.3) |
| IVB | 176 (8.1) | 35 (14.5) | 70 (17.3) | 522 (15.1) | 375 (14.1) | 76 (17.4) | 196 (18.1) |
| BMI categories at index date, n (%) | |  |  |  |  |  |  |
| Moderate/severe thinness | 162 (7.4) | 17 (7.0) | 23 (5.7) | 294 (8.5) | 195 (7.3) | 71 (16.3) | 301 (27.7) |
| Underweight | 176 (8.1) | 10 (4.1) | 33 (8.2) | 326 (9.4) | 244 (9.2) | 45 (10.3) | 146 (13.5) |
| Standard weight | 1388 (63.6) | 165 (68.2) | 253 (62.5) | 2249 (65.0) | 1737 (65.4) | 265 (60.6) | 555 (51.2) |
| Overweight | 390 (17.9) | 40 (16.5) | 83 (20.5) | 519 (15.0) | 420 (15.8) | 51 (11.7) | 78 (7.2) |
| Obesity | 66 (3.0) | 10 (4.1) | 13 (3.2) | 73 (2.1) | 59 (2.2) | 5 (1.1) | 5 (0.5) |
| ECI |  |  |  |  |  |  |  |
| Overall, Mean (SD) | 2.3 (1.3) | 2.4 (1.5) | 2.1 (1.2) | 2.2 (1.4) | 2.1 (1.4) | 2.4 (1.7) | 2.5 (1.9) |
| Median (Min, Max) | 2 [0.0; 10.0] | 2 [0.0; 10.0] | 2 [1.0; 8.0] | 2 [0.0; 12.0] | 2 [0.0; 12.0] | 2 [0.0; 11.0] | 2 [0.0; 11.0] |
| ECI Comorbidities, n (%) | |  |  |  |  |  |  |
| Solid Tumor without Metastasis | 2164 (99.2) | 240 (99.2) | 403 (99.5) | 3393 (98.0) | 2615 (98.5) | 416 (95.2) | 1013 (93.4) |
| Hypertension without complications | 583 (26.7) | 65 (26.9) | 83 (20.5) | 830 (24.0) | 633 (23.8) | 120 (27.5) | 302 (27.8) |
| Peptic Ulcer Disease excluding bleeding | 234 (10.7) | 35 (14.5) | 42 (10.4) | 428 (12.4) | 337 (12.7) | 50 (11.4) | 143 (13.2) |
| Diabetes without complications | 248 (11.4) | 28 (11.6) | 39 (9.6) | 418 (12.1) | 315 (11.9) | 67 (15.3) | 106 (9.8) |
| Liver Disease | 189 (8.7) | 26 (10.7) | 22 (5.4) | 399 (11.5) | 306 (11.5) | 46 (10.5) | 84 (7.7) |
| Fluid and Electrolyte Disorders | 121 (5.6) | 7 (2.9) | 23 (5.7) | 333 (9.6) | 257 (9.7) | 42 (9.6) | 169 (15.6) |
| Cardiac Arrhythmia | 230 (10.5) | 30 (12.4) | 44 (10.9) | 227 (6.6) | 158 (6.0) | 46 (10.5) | 112 (10.3) |
| Chronic Pulmonary Disease | 192 (8.8) | 23 (9.5) | 37 (9.1) | 218 (6.3) | 158 (6.0) | 37 (8.5) | 112 (10.3) |
| Hypothyroidism | 116 (5.3) | 11 (4.6) | 14 (3.5) | 142 (4.1) | 108 (4.1) | 24 (5.5) | 85 (7.8) |
| Congestive Heart Failure | 95 (4.4) | 13 (5.4) | 15 (3.7) | 147 (4.3) | 102 (3.8) | 30 (6.9) | 91 (8.4) |

BMI, body mass index; CRT, chemoradiotherapy; ECI, Elixhauser Comorbidity Index; LA SCCHN, locally advanced squamous cell carcinoma of the head and neck; max, maximum; min, minimum; SD, standard deviation.

**->** indicates “followed by”.

## SUPPLEMENTARY TABLE 4 Demographic and clinical characteristics: By primary tumor location.

| Characteristics | Primary tumor location | | | |
| --- | --- | --- | --- | --- |
|  | Oral cavity | Oropharynx | Hypopharynx | Larynx |
| Age (years, at index-date) | n = 1749 | n = 1925 | n = 1721 | n = 1346 |
| Mean (SD) | 67.2 (14.7) | 64.7 (11.1) | 69.0 (9.6) | 71.0 (9.6) |
| Median (Min, Max) | 70 (20.0, 99.0) | 66 (23.0, 95.0) | 69 (40.0, 94.0) | 71 (31.0, 99.0) |
| Age categories at index-date, n (%) | |  |  |  |
| ≤55 | 355 (20.3) | 413 (21.5) | 148 (8.60 | 82 (6.1) |
| 55–65 | 320 (18.3) | 531 (27.6) | 429 (24.9) | 250 (18.6) |
| 65–75 | 525 (30.0) | 650 (33.8) | 729 (42.4) | 597 (44.4) |
| >75 | 549 (31.4) | 331 (17.2) | 415 (24.1) | 417 (31.0) |
| Gender, n (%) |  |  |  |  |
| Male | 1108 (63.4) | 1543 (80.2) | 1571 (91.3) | 1229 (91.3) |
| Female | 641 (36.6) | 382 (19.8) | 150 (8.7) | 117 (8.7) |
| Follow-up duration (from index-date) | n=1749 | n=1925 | n=1721 | n=1346 |
| Overall, Mean (SD) | 701.6 (604.4) | 868.5 (622.5) | 741.6 (591.2) | 841.4 (611.8) |
| Median (Min, Max) | 553 (1.0, 2399.0) | 744 (1.0, 2421.0) | 609 (2.0, 2427.0) | 720.5 (1.0, 2395.0) |
| Clinical Characteristics |  |  |  |  |
| Index Date, n (%) |  |  |  |  |
| 2016 | 237 (13.6) | 257 (13.4) | 245 (14.2) | 210 (15.6) |
| 2017 | 238 (13.6) | 304 (15.8) | 235 (13.7) | 209 (15.5) |
| 2018 | 308 (17.6) | 354 (18.4) | 301 (17.5) | 244 (18.1) |
| 2019 | 346 (19.8) | 354 (18.4) | 347 (20.2) | 251 (18.7) |
| 2020 | 389 (22.2) | 430 (22.3) | 389 (22.6) | 286 (21.3) |
| 2021 | 231 (13.2) | 226 (11.7) | 204 (11.9) | 146 (10.9) |
| Lymph node involvement, n (%) |  |  |  |  |
| N0 | 570 (32.6) | 150 (7.8) | 206 (12.0) | 728 (54.1) |
| N1 | 362 (20.7) | 701 (36.4) | 302 (17.6) | 190 (14.1) |
| N2 | 679 (38.8) | 910 (47.3) | 947 (55.0) | 357 (26.5) |
| N3 | 137 (7.8) | 164 (8.5) | 266 (15.5) | 71 (5.3) |
| Stage at diagnosis, n (%) |  |  |  |  |
| III | 518 (29.6) | 744 (38.7) | 327 (19.0) | 677 (50.3) |
| IVA | 994 (56.8) | 923 (48.0) | 1072 (62.3) | 588 (43.7) |
| IVB | 237 (13.6) | 258 (13.4) | 322 (18.7) | 81 (6.0) |
| BMI categories at index date, n (%) |  |  |  |  |
| Moderate/severe thinness | 189 (10.8) | 178 (9.3) | 227 (13.2) | 164 (12.2) |
| Underweight | 164 (9.4) | 175 (9.1) | 203 (11.8) | 107 (8.0) |
| Standard weight | 1080 (61.8) | 1186 (61.6) | 1091 (63.4) | 845 (62.8) |
| Overweight | 268 (15.3) | 338 (17.6) | 178 (10.3) | 204 (15.2) |
| Obesity | 48 (2.7) | 48 (2.5) | 22 (1.3) | 26 (1.9) |
| ECI, n (%) |  |  |  |  |
| Overall, Mean (SD) | 2.2 (1.4) | 2.2 (1.5) | 2.3 (1.5) | 2.4 (1.5) |
| Median (Min, Max) | 2 [0.0; 11.0] | 2 [0.0; 12.0] | 2 [0.0; 10.0] | 2 [0.0; 11.0] |
| ECI Comorbidities, n (%) |  |  |  |  |
| Solid Tumor without Metastasis | 1712 (97.9) | 1882 (97.8) | 1671 (97.1) | 1316 (97.8) |
| Hypertension without complications | 421 (24.1) | 450 (23.4) | 462 (26.8) | 385 (28.6) |
| Peptic Ulcer Disease excluding bleeding | 183 (10.5) | 225 (11.7) | 230 (13.4) | 169 (12.6) |
| Diabetes without complications | 190 (10.9) | 203 (10.6) | 186 (10.8) | 193 (14.3) |
| Liver Disease | 127 (7.3) | 217 (11.3) | 191 (11.1) | 143 (10.6) |
| Fluid and Electrolyte Disorders | 140 (8.0) | 191 (9.9) | 185 (10.8) | 110 (8.2) |
| Cardiac Arrhythmia | 149 (8.5) | 130 (6.8) | 153 (8.9) | 137 (10.2) |
| Chronic Pulmonary Disease | 94 (5.4) | 120 (6.2) | 123 (7.2) | 186 (13.8) |
| Hypothyroidism | 67 (3.8) | 76 (4.0) | 114 (6.6) | 86 (6.4) |
| Congestive Heart Failure | 92 (5.3) | 76 (4.0) | 74 (4.3) | 91 (6.8) |

BMI, body mass index; ECI, Elixhauser Comorbidity Index; max, maximum; min, minimum; SD, standard deviation.

## SUPPLEMENTARY TABLE 5 Chemotherapies used for induction prior to surgery and prior to radiotherapy:

## By primary tumor location.

| **Treatment patterns, n (%)** | **Primary tumor location** | | | |
| --- | --- | --- | --- | --- |
| ***Initial chemotherapy: Chemotherapy->Surgery*** | Oral cavity | Oropharynx | Hypopharynx | Larynx |
| n | 49 | 26 | 4 | 12 |
| Fluorouracil + cisplatin | 23 (46.9) | 16 (61.5) | 0 (0.0) | 3 (25.0) |
| Fluorouracil + cisplatin + docetaxel | 16 (32.7) | 6 (23.1) | 0 (0.0) | 4 (33.3) |
| Cetuximab (genetical recombination) + paclitaxel + carboplatin | 4 (8.2) | 1 (3.9) | 0 (0.0) | 1 (8.3) |
| Fluorouracil + nedaplatin | 2 (4.1) | 2 (7.7) | 2 (50.0) | 0 (0.0) |
| Fluorouracil + carboplatin | 0 (0.0) | 1 (3.9) | 2 (50.0) | 2 (16.7) |
| Cisplatin | 3 (6.1) | 0 (0.0) | 0 (0.0) | 1 (8.3) |
| Cetuximab (genetical recombination) + paclitaxel | 1 (2.0) | 0 (0.0) | 0 (0.0) | 1 (8.3) |
| ***Initial chemotherapies: Chemotherapy->Radiotherapy*** |  |  |  |  |
| n | 21 | 121 | 154 | 35 |
| Fluorouracil + cisplatin + docetaxel | 9 (42.9) | 71 (58.7) | 88 (57.1) | 16 (45.7) |
| Fluorouracil + cisplatin | 6 (28.6) | 36 (29.8) | 32 (20.8) | 10 (28.6) |
| Cetuximab (genetical recombination) + paclitaxel + carboplatin | 1 (4.8) | 7 (5.8) | 14 (9.1) | 5 (14.3) |
| Fluorouracil + carboplatin | 0 (0.0) | 2 (1.7) | 6 (3.9) | 0 (0.0) |
| Cisplatin | 1 (4.8) | 0 (0.0) | 4 (2.6) | 1 (2.9) |
| Fluorouracil + docetaxel + nedaplatin | 2 (9.5) | 1 (0.8) | 2 (1.3) | 0 (0.0) |
| Fluorouracil + carboplatin + docetaxel | 1 (4.8) | 1 (0.8) | 1 (0.7) | 1 (2.9) |
| Tegafur/gimeracil/oteracil potassium combination | 0 (0.0) | 1 (0.8) | 0 (0.0) | 1 (2.9) |
| Fluorouracil + cisplatin + cetuximab (genetical recombination) | 0 (0.0) | 0 (0.0) | 1 (0.7) | 1 (2.9) |
| Fluorouracil + nedaplatin | 0 (0.0) | 0 (0.0) | 1 (0.7) | 0 (0.0) |
| Tegafur/uracil | 0 (0.0) | 0 (0.0) | 1 (0.7) | 0 (0.0) |
| Carboplatin + etoposide | 0 (0.0) | 1 (0.8) | 0 (0.0) | 0 (0.0) |
| Cetuximab (genetical recombination) + paclitaxel | 0 (0.0) | 0 (0.0) | 1 (0.7) | 0 (0.0) |
| Fluorouracil | 0 (0.0) | 0 (0.0) | 1 (0.7) | 0 (0.0) |
| Fluorouracil + cisplatin + pembrolizumab (genetical recombination) | 1 (4.8) | 0 (0.0) | 0 (0.0) | 0 (0.0) |
| Cisplatin + etoposide | 0 (0.0) | 0 (0.0) | 1 (0.7) | 0 (0.0) |
| Fluorouracil + cetuximab (genetical recombination) + carboplatin | 0 (0.0) | 1 (0.8) | 0 (0.0) | 0 (0.0) |
| Tegafur/gimeracil/oteracil potassium combination + carboplatin | 0 (0.0) | 0 (0.0) | 1 (0.7) | 0 (0.0) |

**->** indicates “followed by”.

**SUPPLEMENTARY TABLE 6** Healthcare resource utilization in the 12-month post-index period: By primary tumor location

| **Characteristics** |  | **All patients**  N = 5284 | **Primary tumor location** | | | |
| --- | --- | --- | --- | --- | --- | --- |
|  |  |  | **Oral cavity**  N = 1319 | **Oropharynx**  N = 1590 | **Hypopharynx**  N = 1311 | **Larynx**  N = 1064 |
| **Physician visits overall** | N (%) | 4970 (94.1) | 1191 (90.3) | 1525 (95.9) | 1224 (93.4) | 1030 (96.8) |
|  | Mean (SD) | 18.9 (12.5) | 17.7 (13.1) | 19.2 (11.9) | 20.3 (13.0) | 18.1 (12.0) |
|  | Median (Min; Max) | 15 (1; 119) | 14 (1; 88) | 16 (1; 119) | 16 (1; 68) | 14 (1; 67) |
| **All-cause hospital admissions** | N (%) | 5273 (99.8) | 1317 (99.8) | 1584 (99.6) | 1309 (99.8) | 1063 (99.9) |
|  | Mean (SD) | 2.2 (1.6) | 2.1 (1.6) | 2.1 (1.4) | 2.5 (1.7) | 2.1 (1.3) |
|  | Median (Min; Max) | 2 (1; 23) | 1 (1; 23) | 2 (1; 18) | 2 (1; 19) | 2 (1; 12) |
| **All-cause prescriptions** | N (%) | 5273 (99.8) | 1314 (99.6) | 1588 (99.9) | 1309 (99.8) | 1062 (99.8) |
|  | Mean (SD) | 466.4 (410.7) | 437.8 (426.2) | 448 (378.6) | 543.5 (441.1) | 434.5 (386.3) |
|  | Median (Min; Max) | 351 (1; 5221) | 314.5 (2; 5221) | 339 (1; 3253) | 430 (11; 3652) | 318 (5; 3287) |
| **Laboratory tests** | N (%) | 5237 (99.1) | 1292 (98.0) | 1581 (99.4) | 1304 (99.5) | 1060 (99.6) |
|  | Mean (SD) | 37 (33.1) | 33 (31.2) | 35.6 (31.1) | 44.3 (37.1) | 35.2 (31.8) |
|  | Median (Min; Max) | 28 (1; 347) | 25.5 (1; 236) | 26 (1; 219) | 34 (1; 347) | 26 (1; 264) |
| **Diagnostic imaging** | N (%) | 4519 (85.5) | 1104 (83.7) | 1376 (86.5) | 1144 (87.3) | 895 (84.1) |
|  | Mean (SD) | 3.5 (2.1) | 3.7 (2.3) | 3.3 (2.0) | 3.7 (2.1) | 3.4 (2.0) |
|  | Median (Min; Max) | 3 (1; 18) | 3 (1; 18) | 3 (1; 14) | 3 (1; 13) | 3 (1; 15) |
| **Surgeries related to LA SCCHN** | N (%) | 2230 (42.2) | 940 (71.3) | 457 (28.7) | 270 (20.6) | 563 (52.9) |
|  | Mean (SD) | 1.2 (0.4) | 1.1 (0.4) | 1.1 (0.4) | 1.1 (0.4) | 1.2 (0.5) |
|  | Median (Min; Max) | 1 (1; 5) | 1 (1; 4) | 1 (1; 5) | 1 (1; 4) | 1 (1; 4) |
| **Radiation therapy** | N (%) | 3643 (68.9) | 646 (49.0) | 1297 (81.6) | 1050 (80.1) | 650 (61.1) |
|  | Mean (SD) | 34.1 (10.4) | 32.2 (11.9) | 34.6 (10.6) | 34.7 (10.2) | 33.7 (8.4) |
|  | Median (Min; Max) | 35 (1; 146) | 33 (1; 114) | 35 (1; 146) | 35 (1; 88) | 35 (1; 76) |
| **Rehabilitation and homecare** | N (%) | 2912 (55.1) | 871 (66.0) | 728 (45.8) | 736 (56.1) | 577 (54.2) |
|  | Mean (SD) | 22.3 (21.8) | 23.6 (21.2) | 19.7 (20.6) | 23.9 (25.2) | 21.4 (19.2) |
|  | Median (Min; Max) | 17 (1; 241) | 19 (1; 173) | 14 (1; 219) | 17 (1; 241) | 16 (1; 108) |

LA SCCHN, locally advanced squamous cell carcinoma of the head and neck; max, maximum; min, minimum; SD, standard deviation.

**SUPPLEMENTARY TABLE 7** Healthcare costs (JPY, ¥) in the 12-month post-index period: By primary tumor location

| **Characteristics** |  | **All patients**  N = 5284 | **Primary tumor location** | | | |
| --- | --- | --- | --- | --- | --- | --- |
|  |  |  | **Oral cavity**  N = 1319 | **Oropharynx**  N = 1590 | **Hypopharynx**  N = 1311 | **Larynx**  N = 1064 |
| **Physician visits overall** | N (%) | 4970 (94.1) | 1191 (90.3) | 1525 (95.9) | 1224 (93.4) | 1030 (96.8) |
|  | Mean (SD) | 346,183 (346,821.8) | 285,583.6 (350,409.7) | 354,788.7 (341,184.3) | 410,140.4 (372,068.2) | 327,509.7 (303,877.0) |
|  | Total sum of expenditures | 1,720,529,663.0 | 340,130,083.0 | 541,052,711.0 | 502,011,852.0 | 337,335,017.0 |
|  | Cost per patient-year | 123,200.0 | 106,249.5 | 121,666.5 | 150,785.4 | 112,905.2 |
| **All-cause hospital admissions** | N (%) | 5273 (99.8) | 1317 (99.8) | 1584 (99.6) | 1309 (99.8) | 1063 (99.9) |
|  | Mean (SD) | 3,610,720.9 (2,515,486.5) | 3,973,299.1 (2,607,103.3) | 3,187,560 (2,619,950.3) | 4,073,703.8 (2,600,974.2) | 3,221,940.8 (1,892,092.9) |
|  | Total sum of expenditures | 19,039,331,347.0 | 5,232,834,926.0 | 5,049,095,026.0 | 5,332,478,272.0 | 3,424,923,123.0 |
|  | Cost per patient-year | 1,363,327.3 | 1,634,627.4 | 1,135,389.9 | 1,601,674.7 | 1,146,313.1 |
| **All-cause prescriptions** | N (%) | 5273 (99.8) | 1314 (99.6) | 1588 (99.9) | 1309 (99.8) | 1062 (99.8) |
|  | Mean (SD) | 633,549.4 (1,465,043.7) | 611,590.5 (1,067,708.6) | 642,541.7 (2,044,167.0) | 790,678.5 (1,370,098.6) | 453,598.4 (791,373.0) |
|  | Total sum of expenditures | 3,340,705,853.0 | 803,629,972.0 | 1,020,356,236.0 | 1,034,998,132.0 | 481,721,513.0 |
|  | Cost per patient-year | 239,214.0 | 251,037.1 | 229,447.5 | 310,874.3 | 161,231.0 |
| **Laboratory tests** | N (%) | 5237 (99.1) | 1292 (98.0) | 1581 (99.4) | 1304 (99.5) | 1060 (99.6) |
|  | Mean (SD) | 76,010.9 (59,283.6) | 68,267.7 (57,694.7) | 72,689.9 (54,098.8) | 90,184.8 (67,798.7) | 72,965.5 (54,317.9) |
|  | Total sum of expenditures | 398,068,940.0 | 88,201,830.0 | 114,922,710.0 | 117,600,970.0 | 77,343,430.0 |
|  | Cost per patient-year | 28,504.1 | 27,552.4 | 25,842.7 | 35,322.9 | 25,886.7 |
| **Diagnostic imaging** | N (%) | 4519 (85.5) | 1104 (83.7) | 1376 (86.5) | 1144 (87.3) | 895 (84.1) |
|  | Mean (SD) | 5,203.7 (3,686.1) | 5,546.9 (3,789.5) | 4,870.7 (3,520.9) | 5,489.2 (3,888.0) | 4,927.5 (3,472.3) |
|  | Total sum of expenditures | 23,515,600.0 | 6,123,800.0 | 6,702,100.0 | 6,279,600.0 | 4,410,100.0 |
|  | Cost per patient-year | 1,683.9 | 1,912.9 | 1,507.1 | 1,886.2 | 1,476.0 |
| **Surgeries related to LA SCCHN** | N (%) | 2230 (42.2) | 940 (71.3) | 457 (28.7) | 270 (20.6) | 563 (52.9) |
|  | Mean (SD) | 497,112.1 (277,993.6) | 594,830.7 (303,389.3) | 358,968.7 (182,209.0) | 267,215.2 (179,672.1) | 556,344.9 (223,455.3) |
|  | Total sum of expenditures | 1,108,559,900.0 | 559,140,900.0 | 164,048,700.0 | 72,148,100.0 | 313,222,200.0 |
|  | Cost per patient-year | 79,379.4 | 174,663.8 | 36,889.6 | 21,670.6 | 104,834.7 |
| **Radiation therapy** | N (%) | 3643 (68.9) | 646 (49.0) | 1297 (81.6) | 1050 (80.1) | 650 (61.1) |
|  | Mean (SD) | 771,707.7 (302,703.5) | 730,070 (311,972.6) | 817,235.5 (287,646.0) | 782,526.7 (306,130.2) | 704,766.8 (300,373.1) |
|  | Total sum of expenditures | 2,811,331,000.0 | 471,625,200.0 | 1,059,954,400.0 | 821,653,000.0 | 458,098,400.0 |
|  | Cost per patient-year | 201,307.7 | 147,325.8 | 238,351.9 | 246,793.5 | 153,324.4 |
| **Rehabilitation and homecare** | N (%) | 2912 (55.1) | 871 (66.0) | 728 (45.8) | 736 (56.1) | 577 (54.2) |
|  | Mean (SD) | 92,657.8 (127,475.4) | 110,454.9 (171,311.9) | 75,132.6 (90,376.6) | 94,052.6 (121,340.8) | 86,125.2 (88,247.9) |
|  | Total sum of expenditures | 269,819,648.0 | 96,206,180.0 | 54,696,510.0 | 69,222,728.0 | 49,694,230.0 |
|  | Cost per patient-year | 19,320.7 | 30,052.8 | 12,299.6 | 20,791.9 | 16,632.5 |

JPY, ¥, Japanese Yen; LA SCCHN, locally advanced squamous cell carcinoma of the head and neck; max, maximum; min, minimum; SD, standard deviation.
